# Cutaneous Carotenoid Level Measured by Multiple Spatially Resolved Reflection Spectroscopy Sensors Correlates with Vegetable Intake and Is Increased by Continual Intake of Vegetable Juice

**DOI:** 10.3390/diseases9010004

**Published:** 2020-12-31

**Authors:** Hiroki Hayashi, Ikuo Sato, Hiroyuki Suganuma

**Affiliations:** 1 Innovation Division, KAGOME Co., Ltd., 17 Nishitomiyama, Nasushiobara 329-2762, Tochigi, Japan; Hiroyuki_Suganuma@kagome.co.jp; 2 Kurosu Hospital, 2650 Ujiie, Sakura 329-1395, Tochigi, Japan; satou@kurosu-hospital.jp

**Keywords:** carotenoid, vegetable intake, noninvasive measurement, cutaneous carotenoid, cross-sectional study, intervention study

## Abstract

Although vegetables are beneficial for human health, in many countries, the recommended vegetable intake is not reached. To assess vegetable intake, it is important to understand vegetable consumption. Therefore, we conducted a cross-sectional and intervention study of 26 healthy individuals (50% women; 37.0 ± 8.9 years) and estimated vegetable intake on the basis of the cutaneous carotenoid level (CCL) with a noninvasive skin carotenoid sensor, considering that vegetable juice intake can increase CCL. Participants consumed vegetable juice containing 350 g of vegetables daily for 4 weeks. Blood carotenoid levels and CCL were measured for 12 weeks. Cross-sectional analysis showed a significant positive correlation between CCL and vegetable intake (*r* = 0.489). Vegetable juice consumption significantly increased CCL and the blood levels of α-carotene, β-carotene, and lycopene (*p* < 0.05). The correlation coefficient between the blood level and CCL for lycopene was smaller (*r* = 0.001) compared to that between the blood level and CCL for α-carotene (*r* = 0.523) and β-carotene (*r* = 0.460), likely because of bioavailability differences. In conclusion, noninvasive skin carotenoid measurements are effective for determining vegetable intake, and vegetable juice significantly increases CCL.

## 1. Introduction

From a public health perspective, vegetable intake prevents a variety of diseases. Recently, 11 million deaths worldwide were attributed to dietary habits [1]. Typical well-known healthy diets include the Mediterranean and Dietary Approaches to Stop Hypertension (DASH) diets [2,3]. Vegetables are important foods for disease prevention because they contain carotenoids, which are rich in antioxidants, as well as dietary fiber, vitamins, and minerals. In Japan, the government recommended a daily vegetable intake of 350 g; however, the actual intake is 281 g, which is insufficient to maintain optimal health [4]. Despite these recommendations, vegetable intake has not greatly changed over the past decade. This suggests that the government recommendations are inadequate for encouraging vegetable intake.

To improve vegetable intake, it is important to understand intake levels in Japan and predict potential shortages [5]. Although several methods can be used for dietary analyses, including 24 h recall and food frequency questionnaire (FFQ) methods, performing these tests daily is problematic because of their inaccuracy [6]. Thus, we focused on assessing cutaneous carotenoid levels to estimate vegetable intake.

Carotenoids are pigments found mainly in red-, yellow-, and green-colored vegetables; they are absorbed through the intestinal tract and accumulate in various organs [7]. A previous report suggested that carotenoids have antioxidant effects and contribute to the prevention of various diseases [8]. They have a strong ability to remove singlet oxygen when exposed to ultraviolet (UV) radiation in the skin [9]. Carotenoids accumulate in the skin, and a correlation between noninvasive cutaneous carotenoid level (CCL) measurements and vegetable intake has been reported [10]. Previously, the only noninvasive method for measuring carotenoid accumulation in the skin was Raman spectroscopy. Since 2012, Darvin and Meinke (2012, 2013, 2014, 2016) developed a method using light-emitting diodes (LEDs), which is highly correlated with Raman spectroscopy [11,12,13,14]. Meinke (2016, 2017) measured cutaneous carotenoids using multiple spatially resolved reflection spectroscopy (MSRRS) sensors and found that administration of carotenoid-rich antioxidant supplements increased CCLs [14,15].

These reports indicate that a noninvasive cutaneous carotenoid sensor indicates carotenoid levels in the skin, reflecting the intake of carotenoids in vegetables. Few studies examined the relationship between noninvasive CCLs and blood carotenoid levels, and no intervention studies using vegetable juices have been reported [16]. Carotenoids in vegetable juice are absorbed more efficiently than those in raw vegetables [17,18]. Thus, vegetable juice intake is one of the best ways to improve the body antioxidant status. Therefore, an intervention study was conducted to assess the effect of vegetable juice intake on blood carotenoid levels and CCLs. Notably, measuring the effects of vegetable juice intake using a noninvasive cutaneous carotenoid sensor can increase reliability and aid in accurate prediction of vegetables and dietary carotenoid intake. Before the intervention study, a cross-sectional study was conducted to confirm the correlation between CCLs and vegetable intake. Vegetable juice ingestion was found to significantly increase CCL and blood levels of α-carotene, β-carotene, and lycopene (*p <* 0.05). The correlation coefficient between the blood level and CCL for lycopene was smaller (*r* = 0.001) compared to that between the blood level and CCL for α-carotene (*r* = 0.523) and β-carotene (*r* = 0.460). Taken together, noninvasive skin carotenoid measurements are effective for evaluating vegetable intake, and vegetable juice was found to significantly increase CCL.

## 2. Materials and Methods

### 2.1. Study Design

**Figure 1:**
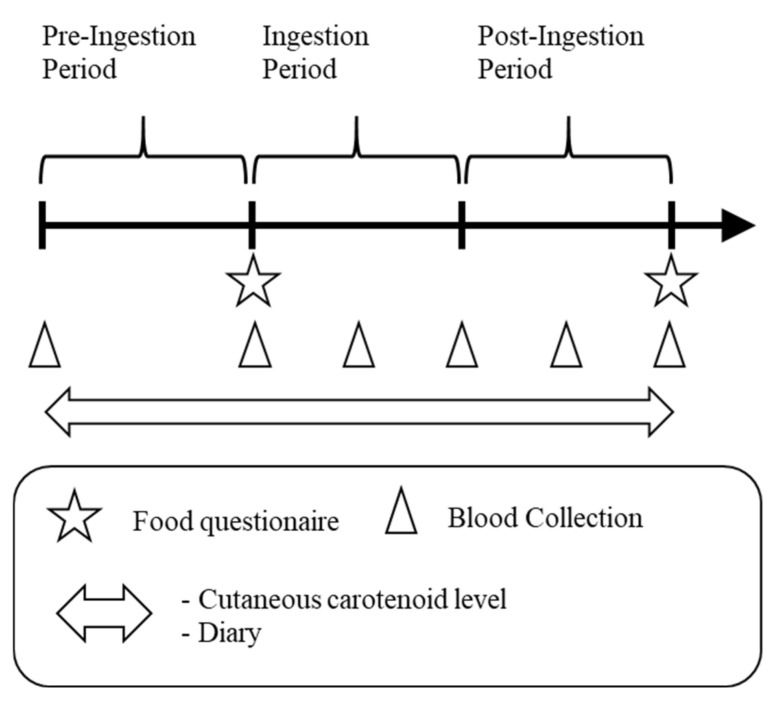
Study schedule. Each period was 4 weeks.

A cross-sectional and intervention study was conducted in healthy adults. The study period was 12 weeks (4 weeks each as the pre-ingestion period, ingestion period, and post-ingestion period) from September to December 2017. Before the intervention study, we analyzed the correlations between blood carotenoid levels and CCLs in a cross-sectional study during the pre-ingestion period. This study was performed in compliance with the Declaration of Helsinki and registered at the UMIN-CTR (University Hospital Medical Information Network-Clinical Trial Registry, UMIN000028320) after approval by the Kagome Research Ethics Review Committee (approval permit number: 2017-R08). The study schedule is shown in Figure 1.

### 2.2. Subjects

The study comprised healthy men and women aged 20 years or older who were living in Japan. The exclusion criteria were as follows: those who habitually consumed 200 mL of vegetable juices or more as the test food, those who were allergic to the test food, those who were hypersensitive to the alcohol-based disinfectant, those who were expected to become pregnant or to begin breastfeed during the study period, and those who participated in other human studies involving the consumption of food high in carotenoids. Twenty-eight subjects were selected for recruitment. Informed consent was obtained from all subjects before starting the study. Subjects were given one carton of vegetable juice daily during the ingestion period. During the 12 week study period, the study subjects were restricted from consuming vegetable juices similar to the test food, foods high in carotenoids, and antioxidant supplements. Vegetables were not restricted if consumed with the daily diet. A diary was kept during the study to monitor health status, food intake, and amount and type of medication used. Our research was approved by the Kagome Research Ethics Review Committee (approval number: 2017-R08).

### 2.3. Test Food

The vegetable juice, “Yasai ichinichi kore ippon”, manufactured by KAGOME CO., LTD. (Nagoya, Japan), was used as the test food. The test juice was a mixture of vegetable juices, including tomatoes, carrots, sprouts, and kale, among others. Each carton had a volume of 200 mL, equivalent to 350 g of fresh vegetables. Therefore, it was more concentrated than squeezed vegetable juices. Carotenoid concentrations in this juice were 6.0 mg/200 mL of α-carotene, 9.8 mg/200 mL of β-carotene, and 16 mg/200 mL of lycopene. The procedure for the analysis of carotenoids was as follows: approximately 5 g of test food was extracted with a 40 mL mixture of hexane, ethanol, acetone, and toluene (10:7:6:7 (*v*/*v*/*v*/*v*)) via ultrasonication for 5 min. Thereafter, the volume of extract was made to 100 mL with ethanol in a volumetric flask. The sample was analyzed by high-performance liquid chromatography using a C18 column. The mobile phase used was acetonitrile, methanol, tetrahydrofuran, and acetic acid (55:40:5:0.1 (*v*/*v*/*v*/*v*)). The detection wavelength was 455 nm for α-carotene and β-carotene and 472 nm for lycopene. The concentration was determined using a standard curve prepared with a high-purity (>90%) carotenoid sample (FUJIFILM Wako Pure Chemical Corporation, Osaka, Japan and Extrasynthese, Lyon Nord, Genay Cedex, France). The nutrients per test food were 69 kcal of energy, 2.4 g of protein, 0 g of lipid, 15.8 g of carbohydrate, and 0–220 mg of sodium. Subjects ingested one portion of the juice every day during the ingestion period. The ingestion time was not specified. During the pre-ingestion and post-ingestion periods, the subjects did not ingest the test food.

### 2.4. Blood Carotenoid Levels

Approximately 10 mL of blood was collected via venipuncture at 09:00 a.m. on the day of blood collection. Six blood samples were collected on day 1 of the pre-ingestion period, day 1 of the ingestion period, day 1 of the third week of the ingestion period, day 1 of the post-ingestion period, day 1 of the third week of the post-ingestion period, and on the last day of the post-ingestion period. The collected blood was clotted by incubating it for 30 min at 20–30 °C, and then the serum was separated by centrifugation (1000× *g*, 5 min) and frozen until analysis. The carotenoid concentrations in the blood were analyzed as described previously [19,20]. The organic solvents, namely, methanol, dichloromethane, and hexane, were mixed thoroughly with the serum, and the organic solvent phase was collected using centrifugation. After volatilization and concentration, the extract was analyzed using high-performance liquid chromatography. Briefly, HPLC was performed on a C30 carotenoid column (YMC, Kyoto, Japan), and the mobile phase used was a mixture of methanol, acetonitrile, *tert*-butyl methyl ether, and water. The concentration was calculated using a standard curve prepared with a high-purity (>90%) carotenoid sample (FUJIFILM Wako Pure Chemical Corporation, Osaka, Japan, and Extrasynthese, Lyon Nord, France) and 8-apo-β-carotenal (Sigma, St. Louis, MO, USA) as the internal standard. The isomers of lycopene were isolated on the basis of the pattern of peaks and retention time on the chromatogram [21].

### 2.5. CCL Measurement

CCLs were measured using an MSRRS sensor manufactured by Biozoom services GmbH (Kassel, Germany). The MSRRS sensor included a plurality of LEDs and a light receiver to measure the CCL. The measurement results were shown as values ranging from 0 to 12, where higher values led to higher carotenoid contents in the skin. The subjects firmly pressed the base of the thumb on the sensor to measure the CCL. During the study period, the CCL of the subjects was measured once per day. The subjects’ CCLs were not measured on the days when they did not come to the office. Additionally, the measurement time was not specific.

### 2.6. Food Questionnaire

To determine the amount of vegetable intake before and after the ingestion period, vegetable intake was assessed twice, on day 1 of the ingestion period and after the post-ingestion period. Vegetable intake was assessed using FFQg (Kenpakusha, Tokyo, Japan), a food intake frequency survey. FFQg is a method for converting daily intake by assessing each food group’s weekly intake frequency [22]. Because the FFQg requires many items other than vegetable intake, such as physical activity, only questions related to vegetable intake were used to reduce the burden on the subjects. Two items were used: frequency of consumption of green and yellow vegetables and frequency of consumption of other vegetables and mushrooms. “Green and yellow vegetables” are defined as vegetables selected by the Ministry of Health, Labor, and Welfare in Japan as containing over 600 μg of carotene per 100 g and dark-colored vegetables. “Other vegetables” include any vegetable other than green and yellow vegetables. The frequency of consumption at breakfast, lunch, and dinner was qualitatively classified as none, little, usually, and plenty. Questionnaires were handed to subjects on paper and collected after completion. Daily intake was converted by automatic calculation from the respective frequency of consumption. Vegetable intake was calculated by adding the intake of green and yellow vegetables and other vegetables and mushrooms.

### 2.7. Statistical Analysis

Statistical analyses were performed using EZR, statistic software based on R and R commander [23]. The correlation between blood carotenoid levels and CCLs was analyzed using Pearson’s correlation coefficient. Significant differences between blood carotenoid levels and CCLs were tested using Dunnett as a post hoc analysis after one-way analysis of variance. A Student’s *t*-test was used to evaluate the significance of differences in FFQg. A risk rate of less than 5% was considered significant.

## 3. Results

### 3.1. Subjects

Twenty-six subjects were included in the analysis after two subjects dropped out voluntarily. The subjects comprised 13 men and 13 women who were aged 37.0 ± 8.9 years, 165.1 ± 7.9 cm in height, and 61.2 ± 11.8 kg in weight (mean ± SD, respectively). Three subjects were administered medicines during the ingestion period but were not excluded because the medications were not thought to directly affect the intended blood carotenoids, CCLs, or body antioxidant status. In addition, two subjects took nutritional supplements during the ingestion period, and one subject occasionally forgot to take the test food. One subject took zinc, and another subject took liver oil as a supplement. Both subjects ingested these supplements throughout the study and, thus, the effect of the intervention could be assessed. Failure to ingest the test food occurred seven times over three weekends. Blood carotenoid levels and CCLs may decrease because of inconsistent ingestion. However, this was the only noncontinuous case. Therefore, it was considered that the CCL of this subject would not be affected, and no exclusion was performed.

### 3.2. Blood Carotenoid Levels

The measured blood carotenoid concentrations are shown in Table 1. No substantial changes were observed before and after the pre-ingestion period. Total carotenoids and α-carotene were significantly increased at week 3 of the ingestion period and day 1, week 3, and the last day of the post-ingestion period compared with day 1 of the ingestion period. β-Carotene significantly increased at week 3 of the ingestion period, day 1 of the post-ingestion period, and week 3 of the post-ingestion period compared with day 1 of the ingestion period. Lycopene significantly increased on day 1 of the post-ingestion period compared with day 1 of the ingestion period. There were differences between isomers; the *cis*-isomer was significantly increased on day 1 and week 3 of the post-ingestion period. In addition, the *trans*-isomer was significantly increased in week 3 of the ingestion period. Other carotenoid levels were not significantly different. The *cis*-isomer lycopene content was significantly higher than that of the *trans*-isomer and gradually decreased after the ingestion period. However, in the post-ingestion period, the *trans*-isomer rapidly decreased to the level measured during the pre-ingestion period.

### 3.3. Cutaneous Carotenoid Level (CCL)

The average percentage of skin measurements over the study period (number of skin measurements in all study subjects) was 77%, excluding days when skin measurements could not be taken because of subject unavailability. Data were compared with weekly means per person. The results showed significantly higher values in the 2–3 weeks of the post-ingestion period compared with week 1 of the ingestion period (Table 2).

### 3.4. Correlation between Blood Carotenoid Levels and CCLs (Cross-Sectional Study)

The correlation between blood total carotenoid concentration and CCL on day 1 of the pre-ingestion period was investigated. A significant positive correlation was found between the two groups, with a correlation coefficient (*R^2^*) of 0.4888. The correlation between blood carotenoid levels and CCLs was significantly positive for α-carotene and β-carotene, with correlation coefficients of 0.748 and 0.793, respectively (Table 3). Other carotenoids showed no significant correlation.

### 3.5. Correlation between Blood Carotenoid Concentrations and CCLs (Intervention Study)

Comparison of the correlation between the magnitude of changes in CCLs and blood carotenoid concentrations from day 1 of the ingestion period revealed significant correlations for α- and β-carotene (Table 4). Other carotenoids were not significantly correlated.

### 3.6. Vegetable Intake

There were no significant differences in the intake of green and yellow vegetables, other vegetables and mushrooms, or their total in the FFQg study (Table 5).

## 4. Discussion

### 4.1. Cross-Sectional Correlation

Serum carotenoids α-carotene and β-carotene showed significant positive correlations with CCLs on day 1 of the pre-ingestion period. Serum carotenoid concentrations were, in descending order, β-carotene, lycopene, lutein, α-carotene, β-cryptoxanthine, and zeaxanthin. Lycopene showed a poor correlation with the CCL, despite showing higher blood levels than that of α-carotene. α-Carotene and β-carotene had similar correlation coefficients and degrees of skin elevation. The two carotenoids were likely absorbed from the intestinal tract and transferred together to the skin via systemic circulation. These results suggest that carotenoids can be transferred to the skin in different ways depending on the type of carotenoid, and that α-carotene and β-carotene easily migrate to the skin. The detection wavelength of the MSRRS sensor used in this study ranged from 458 to 472 nm; Darvin and Meinke also detected carotenoids other than α-carotene and β-carotene [13]. However, when the wavelength is within the range in which lycopene is difficult to detect, it is challenging to detect increased levels in the skin. Although the sensor may include an algorithm for amplifying a portion of the wavelength obtained from the light receiver, the sensor light source is an LED and no specific wavelength can be set, unlike a laser. Therefore, it is unlikely that the low correlation of lycopene was related to the sensor detection wavelength.

### 4.2. Effect of Vegetable Juice Ingestion on Blood Carotenoid Levels

Although the carotenoids found in large amounts in the test food were lycopene, β-carotene, and α-carotene, the increase in blood carotenoid levels of lycopene was lower than that of α-carotene and β-carotene. This may have been caused by the low lycopene bioavailability relative to other carotenoids [24]. The blood concentrations of lycopene isomers differed between the *trans*- and *cis*-isomers, and the blood concentration of the *trans*-isomer rapidly decreased immediately after the ingestion period, whereas the blood concentration of the *cis*-isomer was maintained and gradually decreased. Most of the lycopene exists in the *trans*-form in vegetables and some of the *trans*-form lycopene is exchanged with the *cis*-form during absorption [21]. In bile acid micelles and lipoproteins, lycopene is thought to be stable in the *cis*-form, and the *trans*-form is thought to be in its intermediate state [25]. Thus, the *trans*-form lycopene concentration was increased immediately after absorption, whereas the *cis*-form circulated the body through the liver.

### 4.3. Effect of Vegetable Juice Ingestion on CCLs

There were no significant differences in CCLs during the ingestion period, but there were significant differences in the post-ingestion period. These results show that 4 weeks of vegetable juice ingestion affects the CCL. Carotenoids in vegetable juices are absorbed in the intestinal tract several hours after ingestion and are released into the blood. They accumulate with lipids in organs such as the liver and are then transferred through the blood to various organs by lipoproteins, such as low-density lipoprotein cholesterol [25]. Lademann (2009) suggested that carotenoids are transferred to the skin via sweat and sebum secretion [26]. Similarly, Peytavi (2009) found high levels of lycopene in the skin of the palms and forehead [27]. In 2013, Darvin showed that carotenoids were detected in the top epidermis layer, strongly supporting these theories. Because perspiratory glands mainly mediate lipid secretion onto the skin, carotenoids in the blood appear to be transferred to perspiratory glands along with lipids and are secreted with sebum [13]. The sebum composition is constant, suggesting that the carotenoid concentration in the blood reflects carotenoid concentrations in the sebum. In a study by Meinke (2010), carotenoid intake resulted in a more rapid increase in blood carotenoid levels than in CCL [28]. In this study, after the ingestion period, blood carotenoid levels also decreased more rapidly than the CCL. Although the measurement principles and test systems were not identical and cannot be compared directly, the increase in CCL in this study was slower than that observed by Meinke. The temporal schedule of this study, which was conducted from September to December, may have affected the transfer period, as temperatures were lower and sweating was reduced. Therefore, sweat and sebum secretion was not high. During warmer seasons such as the summer, sebum secretion is increased, and carotenoid transfer from the blood to the perspiratory glands is relatively rapid.

### 4.4. Correlation between Increased CCLs and Individual Blood Carotenoid Levels after Vegetable Juice Ingestion

α-Carotene and β-carotene, which were greatly increased in the blood carotenoid levels, were significantly positively correlated with increased CCL. However, the lycopene content was not significantly correlated. This followed the cross-sectional correlation, suggesting differences in the bioavailability and distribution of α-carotene, β-carotene, and lycopene to the skin.

### 4.5. Comparison with Similar Intervention Studies

Jahns et al. evaluated the correlation between blood carotenoid levels and skin by feeding large amounts of fruits and vegetables [11]. Our results are comparable to those of their study, as we found increased levels of carotenoids in the skin and blood following intervention. This study was compared with that by Meinke because the relationship between carotenoid dose, blood concentration, and the effects of CCLs were similar [28]. Furthermore, increases in carotenoid dose, blood concentration, and CCL were similar to this study (Table 6). The increase in carotenoids was higher in this study than in the study by Meinke. CCLs as total carotenoids were increased by 1.165-fold according to Raman measurement compared to the levels found Meinke [28]. CCL showed a 1.25-fold increase when Meinke used the MSRRS sensor [14]. In this study, the increase in CCL was 1.13-fold. The small increase in vegetable intake was predicted to be caused by the higher standard CCL for Japanese subjects than for Meinke’s study subjects. Nevertheless, a similar level of skin increase was expected. Jung (2014) reported that the intake of vegetables and fruits differs markedly between people in Germany and Korea [29]. People living in Korea consume vegetables and fruits almost daily, whereas one-third of Germans have similar habits. Koreans have significantly higher skin carotenoid levels as measured by MSRRS than Germans and Koreans living in Germany. Although there are no dietary survey data in Jung’s study that can be compared with our results, the frequency of vegetable and fruit consumption in Japanese subjects appears to be high, as in Korean subjects, suggesting that the differences influenced carotenoid levels in the blood and skin.

### 4.6. Advantages of Measuring CCLs

The results indicate that α-carotene and β-carotene, among the carotenoids in vegetable juices, migrate to the skin, whereas lycopene has low bioavailability and is less likely to migrate to the skin. α-Carotene or β-carotene are more efficient than a higher lycopene intake for reducing singlet oxygen damage in the skin. Considering the vegetable juices, carotenoids are more likely to be rapidly transferred to the skin from carrot juice than from tomato juice.

Although CCL measurement is a simple and noninvasive method, it estimates vegetable intake with lower accuracy compared to analysis of blood carotenoid concentrations. Nevertheless, CCL measurements may be useful for approximating vegetable intake and reviewing eating habits.

### 4.7. Limitation of the Study

In this study, the FFQg was used for dietary surveys, but only the portions related to vegetable intake were used. Although the FFQg method does not mention the extraction details, the data may differ from that obtained when vegetable intake is estimated only on the basis of the consumption of food other than vegetables.

This intervention study is a preliminary report because it was conducted on only 26 subjects. A large-scale study is needed to improve the quality of the evidence.

We previously reported that the CCL assessed using the MSRRS sensor correlates significantly with blood carotenoid levels in a cross-sectional study [30]. Nevertheless, this sensor is not suitable for measuring the level of specific carotenoids because of its low wavelength selectivity under current specifications.

## 5. Conclusions

A significant positive correlation was found between CCLs and blood α-carotene and β-carotene concentrations. Subsequently, vegetable juices containing high levels of carotenoids increased blood carotenoid levels and CCLs. A noninvasive cutaneous carotenoid sensor was effective for monitoring the intake of vegetable juices. In addition, for skin protection and health maintenance, carotenoid intake is important, and vegetable juice is an effective means for this purpose. To maintain carotenoid levels in the body and eliminate reactive oxygen from the skin, further studies are needed to evaluate efficient vegetable intake methods, including vegetable juice intake and the use of noninvasive cutaneous carotenoid measuring instruments.

## Figures and Tables

**Table 1:** Blood carotenoid concentration. Mean ± SD (µg/mL) and *p* value (in parentheses).

	Lutein	Zeaxanthin	β-Cryptoxanthin	α-Carotene	β-Carotene	Lycopene			Total Carotenoid
						Total	*cis*-Form	*trans*-Form	
Day 1 of pre-ingestion Period	0.197 ± 0.077	0.039 ± 0.015	0.086 ± 0.048	0.161 ± 0.091	0.462 ± 0.242	0.388 ± 0.131	0.253 ± 0.092	0.138 ± 0.045	1.333 ± 0.383
Day 1 of ingestion period ^1^	0.189 ± 0.061 (0.992)	0.034 ± 0.014 (0.618)	0.088 ± 0.038 (1.000)	0.147 ± 0.128 (0.990)	0.405 ± 0.263 (0.939)	0.371 ± 0.138 (0.991)	0.237 ± 0.084 (0.966)	0.137 ± 0.057 (1.000)	1.232 ± 0.477 (0.919)
Week 3 of ingestion period ^2^	0.190 ± 0.064 (1.000)	0.032 ± 0.015 (0.984)	0.091 ± 0.042 (1.000)	0.378 ± 0.161 *** (<0.001)	0.820 ± 0.393 *** (<0.001)	0.458 ± 0.156 (0.119)	0.285 ± 0.094 (0.248)	0.177 ± 0.068 * (0.045)	1.968 ± 0.679 *** (<0.001)
Day 1 of post-ingestion period ^2^	0.207 ± 0.068 (0.746)	0.038 ± 0.016 (0.757)	0.096 ± 0.048 (0.993)	0.480 ± 0.153 *** (<0.001)	0.946 ± 0.396 *** (<0.001)	0.487 ± 0.158 * (0.022)	0.314 ± 0.105 * (0.021)	0.176 ± 0.062 (0.051)	2.253 ± 0.587 *** (<0.001)
Week 3 of post-ingestion period ^2^	0.197 ± 0.063 (0.978)	0.034 ± 0.017 (0.999)	0.092 ± 0.051 (0.999)	0.306 ± 0.109 *** (<0.001)	0.657 ± 0.322 * (0.016)	0.452 ± 0.164 (0.168)	0.311 ± 0.115 * (0.028)	0.144 ± 0.054 (0.975)	1.738 ± 0.507 ** (0.001)
Last day of post-ingestion period ^2^	0.199 ± 0.346 (0.942)	0.036 ± 0.362 (0.924)	0.147 ± 0.387 (0.079)	0.236 ± 0.208 * (0.021)	0.553 ± 0.333 (0.261)	0.411 ± 0.227 (0.741)	0.281 ± 0.243 (0.311)	0.132 ± 0.308 (0.997)	1.583 ± 0.384 * (0.042)

^1^ Comparison to the day 1 of pre-ingestion period, ^2^ Comparison to the day 1 of ingestion period; * *p <* 0.5, ** *p <* 0.1, *** *p <* 0.001.

**Table 2:** Cutaneous carotenoid level.

Period	Week	Cutaneous Carotenoid Level
Pre-ingestion	1	6.55 ± 0.88
	2	6.69 ± 0.98
	3	6.62 ± 0.89
	4	6.64 ± 0.96
Ingestion	1	6.63 ± 1.02
	2	6.71 ± 0.96
	3	6.91 ± 0.96
	4	7.16 ± 1.00
Post-ingestion	1	7.34 ± 1.02
	2	7.50 ± 0.93 *
	3	7.46 ± 0.93 *
	4	7.23 ± 0.91

Results are presented as the mean ± SD of the week, *n* = 26. * *p <* 0.05 vs. week 1 of ingestion period.

**Table 3:** Correlation coefficient between the cutaneous and blood carotenoid levels in cross-sectional analysis, day 1 of the pre-ingestion period.

	Correlation Coefficient
Lutein	0.206
Zeaxanthin	0.074
β-Cryptoxanthin	0.080
α-Carotene	0.748 **
β-Carotene	0.793 **
Lycopene	0.065

Results were analyzed by Pearson’s correlation analysis, *n* = 26. ** *p <* 0.01.

**Table 4:** Correlation coefficients between cutaneous carotenoid level and blood carotenoid concentration changes from day 1 of the ingestion period.

	Day 1 of Ingestion Period	Week 3 of Ingestion Period	Day 1 of Post-Ingestion Period	Week 3 of Post-Ingestion Period	Last Day of Post-Ingestion Period
Lutein	0	0.077	−0.059	−0.104	−0.034
Zeaxanthin	0	−0.064	−0.277	−0.242	−0.109
β-Cryptoxanthin	0	0.119	−0.208	0.121	0.007
α-Carotene	0	0.420 *	0.521 **	0.523 **	0.436 *
β-Carotene	0	0.398 *	0.46 *	0.325	0.134
Lycopene	0	0.001	−0.017	−0.129	−0.189
*cis*-Lycopene	0	−0.192	−0.157	−0.170	−0.196
*trans*-Lycopene	0	0.192	0.132	−0.055	−0.139

Results were analyzed by Pearson correlation analysis, *n* = 26. * *p <* 0.05, ** *p <* 0.01 vs. day 1 of the ingestion period.

**Table 5:** Vegetable intake estimated via food questionnaires.

	Pre-Ingestion Period	Post-Ingestion Period	Difference
Green and yellow vegetables (g)	79.5 ± 53.2	80.6 ± 39.0	1.1 ± 30.2
Other vegetables (g)	139.6 ± 83.7	124.0 ± 55.0	−15.6 ± 55.3
Total vegetables (g)	219.1 ± 132.2	204.6 ± 89.8	−14.5 ± 73.5

Results are presented as the mean ± SD, *n* = 26.

**Table 6:** Comparison between Meinke’s study and this study.

	Meinke’s Study [28]			This Study		
	Ingestion Period (Days)					
	28 Days			28 Days		
	Amount of Carotenoids in Test Food (mg)					
**Lutein**	9			-		
**α-Carotene**	-			6		
**β-Carotene**	3			9.8		
**Lycopene**	0.78			16		
	**Blood Carotenoids Concentration (Mean, µg/mL)**					
	Pre	Post	Difference	Pre	Post	Difference
**β-Carotene**	0.172	0.278	0.106	0.462	0.946	0.484
**Total Carotenoids**	0.534	0.792	0.258	1.232	2.253	1.022
***cis*-Lycopene**	0.0432	0.0553	0.0121	0.237	0.314	0.077
	**Increased Blood Carotenoid Concentration per Ingested Carotenoids (Mean, µg/mL per mg Ingestion)**					
**β-Carotene**	0.0353			0.0494		
**Lycopene**	0.0202			0.0321		

## Data Availability

Data sharing not applicable.
